# Dupilumab efficacy in children aged 6 months to younger than 6 years with moderate-to-severe atopic dermatitis and clinical characteristics of early super-responders

**DOI:** 10.1186/s12887-026-07381-6

**Published:** 2026-07-22

**Authors:** Li Ma, Mengjun Wei, Wenjia Mou, Li Xia, Nan Yu, Miaomiao Zhao, Peiyao Yu, Xiaoyu Jia, Yuanyuan Shang

**Affiliations:** Department of Dermatology, General Hospital of Ningxia Medical University, Yinchaun, 750001 China

**Keywords:** Atopic dermatitis, Dupilumab, Children, Predictive factors, Efficacy, Safety

## Abstract

**Background:**

To evaluate the efficacy of dupilumab in treating moderate-to-severe atopic dermatitis (AD) in children aged 6 months to 6 years and to investigate the clinical characteristics influencing early super-responders（eSR).

**Methods:**

This study adopted a prospective design and enrolled pediatric patients with moderate-to-severe AD who were treated at the Department of Dermatology, General Hospital of Ningxia Medical University, from January 2023 to March 2025. In accordance with the patients’ age and body weight, a subcutaneous injection of dupilumab (200–300 mg) was administered every 4 weeks. At baseline, week 4, week 12, and week 16 of treatment, the efficacy of dupilumab and the family quality of life were evaluated using the following tools: the Eczema Area and Severity Index (EASI), Investigator’s Global Assessment (IGA), Peak Pruritus Numeric Rating Scale (PP-NRS), Children’s Dermatology Life Quality Index (CDLQI)/Infant’s Dermatology Life Quality Index (IDQoL), and Dermatitis Family Impact (DFI). Improvements in comorbid conditions were assessed using the Rhinoconjunctivitis Control Assessment Test (RCAT), Child Asthma Control Test (C-ACT), and Oral Food Challenge (OFC). Moreover, adverse events that occurred during the treatment period were recorded systematically. The proportions of patients who achieved EASI75 (≥75% improvement in EASI score from baseline), a ≥4-point reduction in PP-NRS, and a ≥4-point improvement in CDLQI / IDQoL concurrently at week 4, as well as those who met both EASI75 and IGA 0/1(clear/almost clear) at week 16, were documented. Linear mixed models were used to analyze changes in clinical scores at different time points before and after dupilumab treatment, and logistic regression was applied to identify factors influencing eSR to dupilumab treatment.

**Results:**

A total of 50 children with moderate to severe AD, including 21 males (42.00%) and 29 females (58.00%), aged 4.24±1.81, were included in this study. Clinical scores related to skin lesions and pruritus were significantly lower at weeks 4, 12, and 16 postdupilumab treatment than at baseline (*P*< 0.001). At week 4, 40.00% (20/50) of patients achieved the primary efficacy endpoints. By week 16, 87.10% (27/31) and 90.32% (28/31) of patients had EASI75 and an IGA 0/1, respectively. The proportion of patients with severe/moderate DFI scores decreased markedly from 100% at baseline to 9.68% (*P* < 0.001). Notably, the prevalence of recurrent upper respiratory tract infections decreased significantly (P = 0.017), while the improvement rates for allergic rhinitis and food allergies reached 84.21% (*P* < 0.001) and 84.61% (*P* < 0.001), respectively. Multivariate logistic regression revealed that a high absolute basophil count (OR = 0.104, 95% CI = 0.021-0.506, *P* = 0.005) and the presence of allergic rhinitis (OR = 0.223, 95% CI = 0.055-0.896, *P* = 0.035) were independent predictors of eSR. Receiver operating characteristic (ROC) curve analysis demonstrated high predictive accuracy (AUC = 0.773; *P* = 0.001), indicating that a baseline absolute basophil count less than 0.035×109/L was associated with a better eSR to dupilumab. No serious adverse events were observed during treatment.

**Conclusion:**

Dupilumab demonstrates significant efficacy and a favorable safety profile in the treatment of moderate-to-severe atopic dermatitis in children aged 6 months to 6 years. During treatment, patients presented alleviated allergic comorbidities, decreased incidence of recurrent upper respiratory tract infections, and improved quality of life for children and their families. Elevated absolute basophil count in peripheral blood and the presence of allergic rhinitis were independent predictors of eSR to dupilumab in children with AD.

**Supplementary Information:**

The online version contains supplementary material available at https://doi.org/10.1186/s12887-026-07381-6.

## Background

Pediatric atopic dermatitis (AD) is a chronic inflammatory and pruritic skin disease that is often accompanied by skin barrier dysfunction and immune dysregulation (dominant Th2-type inflammation). The global prevalence of AD in the pediatric population is as high as 15% to 20% [1], and in China, the prevalence among infants under 1 year of age is 30.48%—which is the among all age groups [2]. Moderate-to-severe AD not only elicits severe pruritus, skin erosion and other clinical manifestations but also significantly impairs children’s sleep quality, growth and development, and overall quality of life. Thus, AD imposes a substantial burden on family caregiving [3, 4]. AD is the initiating event of the atopic march, and approximately 10% of children with AD are at risk of progressing along this allergic trajectory [5]; this risk is further elevated by moderate-to-severe eczema, early-onset eczema, or persistent eczema [6]. For pediatric patients with moderate-to-severe AD who have an inadequate response or intolerance to conventional therapies (topical corticosteroids and calcineurin inhibitors) and are limited by the serious adverse events associated with systemic immunosuppressants, the development and clinical application of biologic agents represent a breakthrough in therapeutic management. Dupilumab, a fully human monoclonal antibody targeting interleukin-4 receptor alpha (IL-4Rα), blocks the release of inflammatory mediators from type 2 inflammation pathways. It has been approved in China for children aged > 6 months with moderate-to-severe AD [7]. While clinical trials worldwide have demonstrated the efficacy and safety of dupilumab in significantly improving the signs, symptoms and quality of life in children with AD, real-world evidence regarding its effectiveness and safety in the Chinese pediatric population aged 6 months to 6 years remains scarce [8]. Furthermore, no studies have reported on the long-term benefits of dupilumab for improving allergic comorbidities and family quality of life in this cohort, or on the clinical factors influencing early super-responders (eSR) to the agent [9].

This study evaluated the efficacy and safety of dupilumab in children aged 6 months to 6 years with moderate-to-severe AD who were treated at the Department of Dermatology, General Hospital of Ningxia Medical University. Additionally, we analyzed clinical characteristics that predict eSR 4 weeks after the first subcutaneous injection of dupilumab.

## Materials and methods

### Eligibility of participants

A prospective, active monitoring study was conducted for pediatric moderate-to-severe AD patients who received dupilumab at least once between January 2023 and March 2025 at the Department of Dermatology, General Hospital of Ningxia Medical University.

The inclusion criteria were as follows: (1) age 6 months to 6 years; (2) a diagnosis of AD according to the Hanifin–Rajka criteria [10]; (3) an Eczema Area and Severity Index (EASI) score >7; (4) an Investigator Global Assessment (IGA) score ≥3 at baseline; (5) a SCORing Atopic Dermatitis (SCORAD) score ≥25; (6) lesional involvement of body surface area (BSA) ≥10%; (7) resistance or intolerance to conventional therapies (including topical corticosteroids, systemic medications, etc.) or unwillingness to continue conventional treatment; and (8) willingness to participate in the study and undergo follow-up.

The exclusion criteria were as follows: (1) allergy to dupilumab or any of its components; (2) severe organ dysfunction (e.g., heart, liver, or kidney), concurrent hematological diseases, or tumors; (3) incomplete clinical data that may affect efficacy evaluation; (4) a history of prior biologic agent use in patients and (5) other mental disorders preventing cooperation with data collection. All patient data were collected in the presence of guardians, and written informed consent was obtained from all guardians.

This study was approved by the Medical Research Ethics Committee of the General Hospital of Ningxia Medical University (Ethics Approval No: KYLL-2024-1447) and adhered to the provisions of the Declaration of Helsinki. This study was funded by the Ningxia Natural Science Foundation Key Project (Grant number: 2023AAC02061).

### Study design and data collection

Pediatric patients were treated with dupilumab injection (300 mg/2.0 ml/vial or 200 mg/1.14 ml/vial prefilled syringe; Sanofi Winthrop Industrie) administered subcutaneously. During treatment, all the children used moisturizers concurrently, and topical agents were allowed as adjunctive therapy. Clinical data were collected at baseline and at follow-up visits at 4, 12, and 16 weeks of treatment. Blood samples were collected before baseline treatment to measure complete blood count (CBC) and total immunoglobulin E (IgE) levels.

We assessed AD severity, comorbidity control (allergic rhinitis, asthma, and food allergy), and quality of life using the following validated tools at baseline and at 4, 12, and 16 weeks post-injection: the SCORAD, EASI, IGA, Peak Pruritus Numerical Rating Scale (PP-NRS), Patient-Oriented Eczema Measure (POEM), Children’s Dermatology Life Quality Index (CDLQI)/Infant Dermatitis Quality of Life Index (IDQoL), Atopic Dermatitis Control Tool (ADCT) [11], Rhinitis Control Assessment Test (RCAT), Childhood Asthma Control Test (C-ACT), and Oral Food Challenge (OFC) [12–14]. Caregivers’ quality of life was evaluated using the Dermatitis Family Impact (DFI) index [15].

Recurrent upper respiratory tract infections (rURTIs) were defined as ≥ 1 episode per month from September to April of the following year ([16–17]). Extrinsic AD was defined as a total serum IgE concentration > 15 IU/mL (< 1 year old), > 60 IU/mL (1–5 years old), or > 90 IU/mL (6 years old) combined with a personal or family history of allergic disease or positive allergen-specific IgE; all other cases of AD were classified as instrinsic AD [18].

### Reaction evaluation

Patient disease severity was assessed using the SCORAD, which ranges from 0 to 103 points. Moderate AD was defined as a score of 25–50, and severe AD was defined as a score of 51–103. Quality of life was evaluated using the CDLQI/IDQoL. The ADCT has a total score of 0–24, with scores < 7 indicating disease control. The RCAT was used to assess allergic rhinitis remission over the past week, with patients scoring ≥ 22 deemed to have “symptom control” [12]. The C-ACT is used to evaluate asthma control in children, with improvement defined as a score ≥ 20 over the past four weeks [19]. Desensitization efficacy for food allergens was evaluated strictly following the standardized procedures outlined in the Expert Consensus on Standardized Processes for OFC [20]. A key judgment criterion was improvement in food allergy, which was defined as a negative target food challenge during a standardized OFC test (i.e., if no allergic reaction occurred). The DFI scale, which is used to assess Caregivers’ quality of life, ranges from 0 to 30 points. The DFI scores ranged from 0 to 5 (normal), 6 to 10 (mild impact), 11 to 20 (moderate impact), and 21 to 30 (severe impact) [21].

Patients who concurrently achieved EASI75(≥ 75% improvement in EASI score from baseline), a ≥ 4-point improvement in PP-NRS score, and a ≥ 4-point improvement in CDLQI/IDQoL score at week 4 - defined as eSR [9]. Additionally, the proportions of patients who met EASI50, EASI75, EASI90, IGA 0/1, a ≥ 4-point improvement in PP-NRS score, a ≥ 4-point improvement in the CDLQI/IDQoL, or an ADCT < 7 at week 16 were documented and analyzed. Adverse reactions (ADRs) during treatment were recorded.

### Statistical analysis

The data were analyzed using IBM SPSS version 27.0. Categorical variables are expressed as numbers (n) and percentages (%), and normally distributed continuous variables are expressed as the mean ± standard deviation (SD) or least-square mean (LSM) with standard error (SE). Linear mixed models (LMMs) were employed to analyze changes in clinical scores across different time points before and after dupilumab treatment, with time set as the fixed effect and individual patients as the random effect to correct for within-subject correlation and handle missing data during follow-up. The restricted maximum likelihood (REML) method was used to estimate the variance components of the model. The LSM and 95% confidence intervals (CIs) at each time point were derived from the model, and pairwise comparisons were adjusted using the Bonferroni method with a corrected alpha level of α’ = 0.05/5 = 0.01. The chi-square (χ2) test was used to analyze the proportions of patients who achieved IGA 0/1, EASI50, EASI75, EASI90, a ≥ 4-point improvement in CDLQI/IDQoL, a ≥ 4-point improvement in PP-NRS, and ADCT < 7 at weeks 4, 12 and 16. For pairwise multiple comparisons in the χ2 test, the Bonferroni method was used to correct the test level with α’ = 0.05/3 = 0.017. Multicollinearity was assessed using variance inflation factors (VIF), variables with VIF > 10 were removed. Univariate logistic regression was used to screen variables with *P* < 0.2, which were then included in multivariate logistic regression to calculate the odds ratio (OR), 95% confidence interval (CI), adjusted OR, and adjusted 95% CI for eSR. Based on the events-per-variable (EPV) ≥ 10 principle, predictor variables were selected to reduce overfitting, and parallel bootstrap internal validation was performed to assess the robustness of the regression coefficients. Natural logarithm (Ln) transformation was applied to resolve data overflow issues in the raw data calculations, ensuring robust results. Baseline absolute basophil count was used to construct a receiver operating characteristic (ROC) curve; the Youden index was employed to determine the optimal cut-off, and corresponding sensitivity, specificity, and area under the curve (AUC) were reported. *P* < 0.05 was regarded as statistically significant.

## Results

### Baseline demographic characteristics

**Fig. 1:**
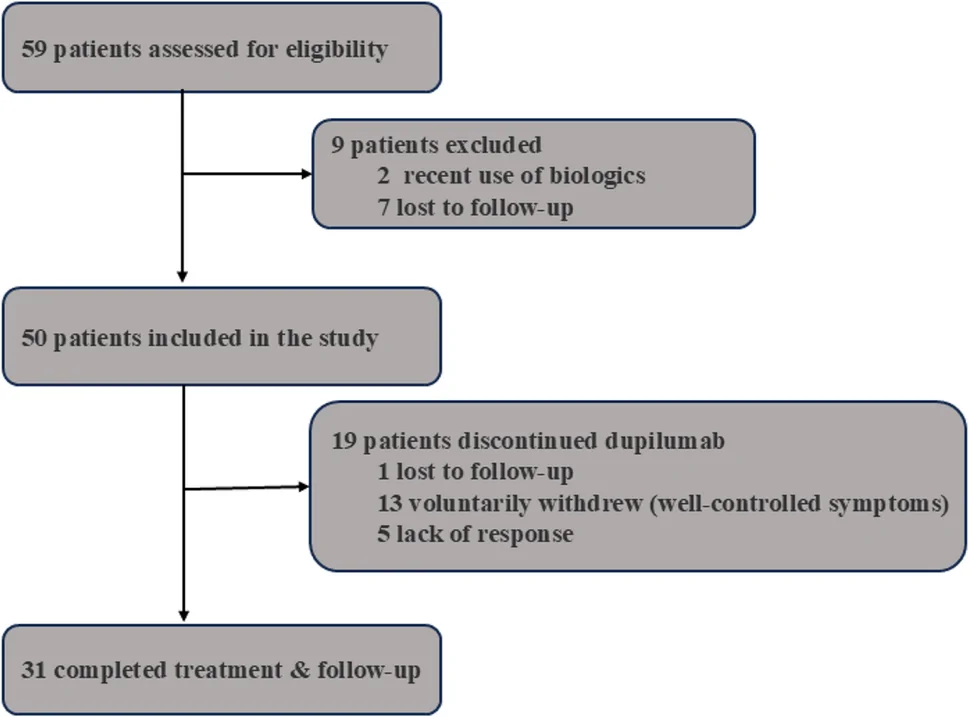
Flow chart of recruitment of potential patients to the study

In accordance with the inclusion and exclusion criteria, a total of 59 children with moderate-to-severe AD aged 6 months to 6 years were enrolled from January 2023 to March 2025. Nine patients with prior biologic use or who were lost to follow-up were excluded. Ultimately, 50 patients who received dupilumab injections and who completed follow-up were included, among whom 31 completed the 16-week follow-up. While 19 did not complete the full follow-up, yielding a dropout rate of 38.00%. The reasons for dropout were as follows: (1) 1 patient lost to follow-up; (2) 5 patients lack of response; and (3) 13 patients withdrew voluntarily due to well-controlled symptoms. (Fig. 1).

Among the 50 children with AD, 19 (38.0%) were assigned to receive 200 mg dupilumab every 4 weeks, and 31 (62.0%) were assigned to receive 300 mg dupilumab every 4 weeks. A total of 50, 38 and 31 patients completed the injections and subsequent follow-up at week 4, week 12 and week 16, respectively. A total of 31 children completed at least 4 injections, including 22 (71.0%) in the 300 mg group and 9 (29.0%) in the 200 mg group. Their demographic and baseline clinical characteristics are summarized in Table 1.

**Table 1:** Demographic characteristics of 50 patients using dupilumab and their caregivers [(x̅ ± s/median (IQR) /(n,%)])

Baseline characteristics	Total	Baseline characteristics	Total
Gender		SCORAD	58.98 ± 15.05
Male	21(42.00)	EASI	15.32 ± 5.50
Female	29(58.00)	IGA	3.30 ± 0.46
Age (years)	4.24 ± 1.81	POEM	13.08 ± 6.20
Onset ≤ 6 months of age	26(52.00)	CDLQI/IDQoL	13.80 ± 5.97
Molecular endotype		PP⁃NRS	7.28 ± 1.67
intrinsic AD	14(28.00)	ADCT	17.22 ± 4.29
extrinsic AD	36(72.00)	DFI	19.96 ± 3.85
Moderate AD	16(32.00)	Previous Topical Therapies	
Severe AD	34(68.00)	TCS	50(100.00)
rURTIs	30(60.00)	PDE-4 inhibitor	21(42.00)
Allergic Comorbidities	31(62.00)	TCI	3 (6.00)
AR	23(46.00)	Previous Systemic Therapies	
Asthma	3(6.00)	Antihistamines	50(100.00)
FA	16(32.00)	Systemic Corticosteroids	3(6.00)
Urticaria	2(4.00)	Oral Chinese Medicine	1(2.00)
Allergic Conjunctivitis	3(6.00)	Laboratory parameters	
Family History of Atopy	28(56.00)	Total sIgE level (IU/ml)	159.50 (34.8, 611.50)
AR	24(48.00)	AEC(×10⁹/L)	0.30(0.18, 0.66)
Eczema	10(20.00)	ABC(×10⁹/L)	0.04(0.03, 0.06)
Breastfeeding	41(82.00)	MCV(fL)	81.91 ± 3.48

Data are median (IQR), n (%), or mean (SD). Unless otherwise indicated, the table shows data for the full analysis set

*TCS *Topical corticosteroids, *PDE-4* inhibitor Phosphodiesterase Inhibitors, *TCI* Topical Calcineurin Inhibitors, *sIgE* serum immunoglobulin E, *AEC* Absolute Eosinophil Count, *ABC *Absolute basophil count, *MCV *Mean Corpuscular Volume

Among the 50 patients, the mean (SD) age was 4.24 ± 1.81 years. AD was predominantly ​extrinsic (72.00%). Thirty-one children (62.00%) had allergic comorbidities, 30 (60.00%) experienced rURTIs, and all caregivers reported moderate or greater impairment in quality of life. Half (50.00%) of the children had elevated absolute basophil counts (>0.07 × 10⁹/L). Prior to dupilumab treatment, all the children had received topical corticosteroids and antihistamines, and 6% had received oral corticosteroids systemically. The details are shown in Table 1. Table 2 compares the baseline clinical characteristics between completers of the 16-week follow-up and dropouts.

**Table 2:** Demographic characteristics of 50 patients by 16-week follow-up completion status [(x̅ ± s/median (IQR) /(n,%)])

Baseline characteristics	Completed follow-up (*n* = 31)	Incomplete follow-up (*n* = 19)	Statistic	*P* value
Male	13(41.94)	8(42.11)	χ2 = 0.00	0.991
Female	18(58.06)	11(57.89)		
Age (years)	4.39 ± 1.94	3.99 ± 1.60	t = 0.77	0.427
Onset ≤ 6 months of age	19(61.29)	7(36.84)	χ2 = 2.82	0.093
Moderate AD	9(29.03)	7(36.84)	χ2 = 0.33	0.566
Severe AD	22(70.97)	12(63.16)		
extrinsic AD	22(70.97)	14(73.68)	χ2 = 0.04	0.836
rURTIs	24(77.42)	6(31.58)	χ2 = 10.31	0.001
Allergic Comorbidities				
AR	19(61.29)	4(21.05)	χ2 = 7.68	0.006
Asthma	2(6.45)	1(5.26)	χ2 = 0.03	0.864
FA	12(38.71)	4(21.05)	χ2 = 1.69	0.194
Breastfeeding, n (%)	25(80.65)	16(84.21)	χ2 = 0.10	0.750
Baseline SCORAD	61.14 ± 16.29	55.44 ± 12.37	t = 1.31	0.209
Baseline EASI	15.84 ± 6.01	14.48 ± 4.58	t = 0.84	0.249
Baseline IGA	3.32 ± 0.48	3.26 ± 0.45	t = 0.44	0.371
Baseline POEM	13.84 ± 6.89	11.84 ± 4.79	t = 1.11	0.029
Baseline CDLQI/IDQoL	13.58 ± 6.01	14.16 ± 6.06	t = -0.33	0.658
PP⁃NRS	7.65 ± 1.72	6.68 ± 1.42	t = 2.04	0.142
ADCT	17.84 ± 4.30	16.21 ± 4.18	t = 1.31	0.815
DFI	19.74 ± 4.19	20.32 ± 3.28	t = -0.51	0.206
Baseline IgE	157.00(34.80, 457.00)	182.00(34.80, 766.00)	Z = -0.69	0.490
AEC(×10⁹/L)	0.28(0.19, 0.70)	0.32(0.16, 0.42)	Z = -0.85	0.396
ABC(×10⁹/L)	0.04(0.03, 0.06)	0.04(0.03, 0.06)	Z =-0.10	0.919
Hemoglobin	131.06 ± 9.70	131.58 ± 9.15	t = -0.19	0.757
MCV(fL)	81.50 ± 3.23	82.57 ± 3.85	t = -1.05	0.132
MCH(pg)	27.33 ± 1.43	27.55 ± 1.14	t = -0.58	0.710

Categorical variables are presented as frequencies and percentages [n (%)], and between-group comparisons were performed using χ² test. Normally distributed continuous variables are expressed as mean ± standard deviation (SD), and differences between groups were compared using the independent-samples t-test. Non-normally distributed continuous variables are described as median (IQR), and the Wilcoxon rank-sum test was used for intergroup comparisons. *P* < 0.05

*MCH *Mean Corpuscular Hemoglobin

### Efficacy and changes in clinical scores

**Fig. 2:**
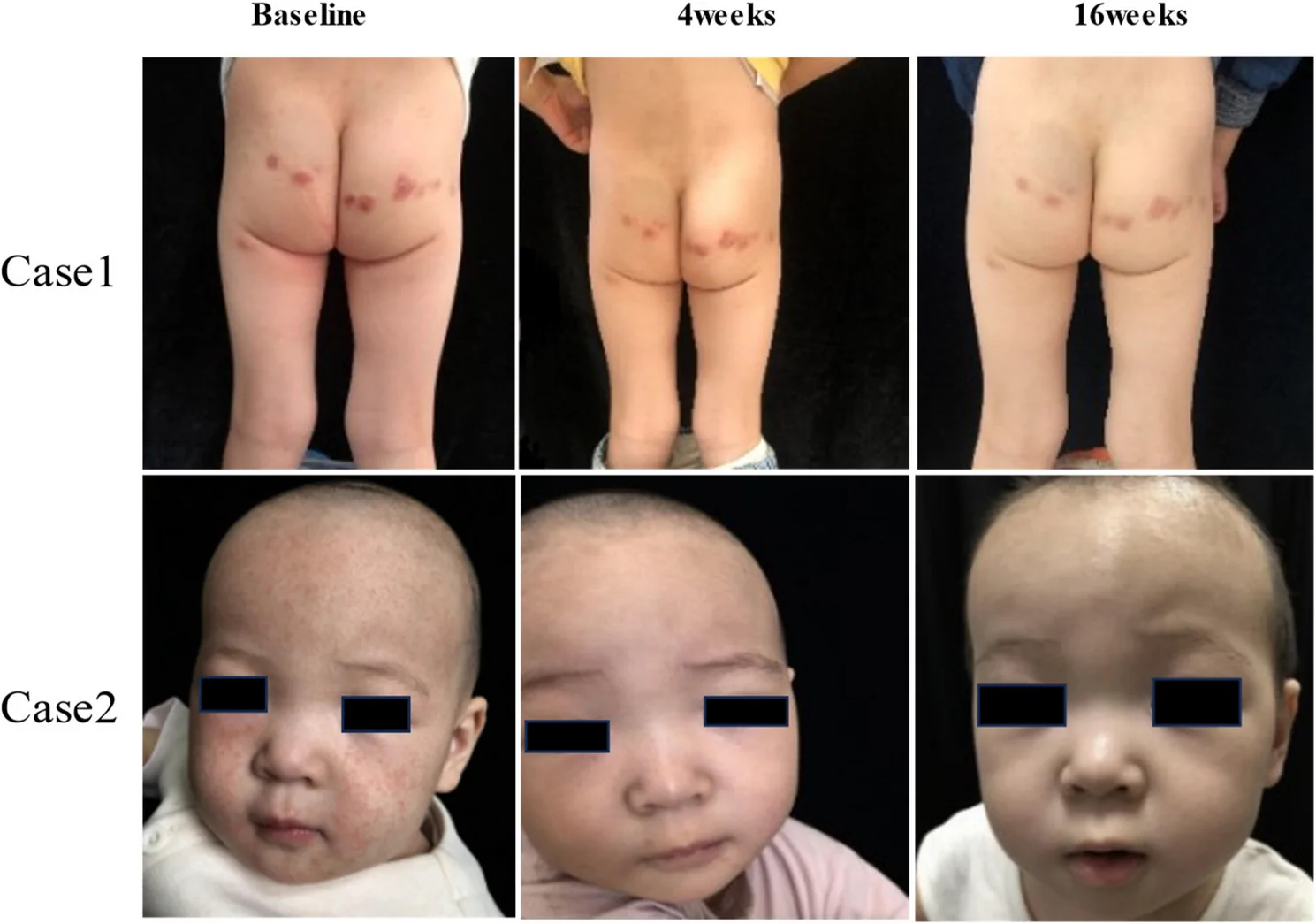
Lesional manifestations of children with AD before dupilumab treatment and at weeks 4/16 post-treatment​. Case 1: A 4-year-1-month-old male presented with erythematous papules and erosive exudation on the buttocks; marked improvement in buttock erosions/exudation was observed after 16 weeks of treatment. Case 2: A 1-year-2-month-old female had diffusely distributed, millet-sized erythematous papules (partially confluent) on the face; no obvious facial lesions remained after 16 weeks of treatment

At weeks 4, 12, and 16 of dupilumab treatment, compared with the baseline scores, the SCORAD, EASI, IGA, POEM, CDLQI/IDQoL, PP-NRS, ADCT, and DFI scores significantly improved (*P* < 0.001). (Table 3). The improvement in skin lesions before and after treatment is shown in Fig. 2.

**Table 3:** Changes in efficacy, and family impact scores (mean ± SD) in children with atopic dermatitis (AD) before dupilumab treatment and at weeks 4, 12, and 16 post-treatment

Scoring Scale	Baseline (50)	4 weeks (50)	12 weeks(38)	16 weeks (31)	F	*P*
SCORAD	58.98 ± 2.27	34.11 ± 2.98^ab^	22.10 ± 2.44^ab^	16.25 ± 2.29^ac^	86.343	< 0.001
EASI	15.32 ± 0.78	6.46 ± 0.80^ab^	2.76 ± 0.51^ab^	1.59 ± 0.31^ac^	130.042	< 0.001
IGA	3.30 ± 0.10	1.42 ± 0.18^ab^	0.84 ± 0.14^ab^	0.49 ± 0.14^ac^	181.170	< 0.001
POEM	13.08 ± 3.23	6.34 ± 3.19^ab^	3.86 ± 3.15^ac^	2.97 ± 3.13^ac^	45.578	< 0.001
CDLQI/IDQoL	13.80 ± 0.85	7.24 ± 1.02^ab^	4.31 ± 0.61^ac^	3.79 ± 0.63^a^	40.219	< 0.001
PP⁃NRS	7.28 ± 0.36	3.98 ± 0.43^ab^	2.90 ± 0.45^ac^	2.00 ± 0.47^a^	64.916	< 0.001
ADCT	17.22 ± 3.84	8.24 ± 3.92^ab^	4.96 ± 3.86^ac^	4.08 ± 3.87^a^	92.947	< 0.001
DFI	19.96 ± 0.76	14.18 ± 1.16^ab^	10.00 ± 1.34^ac^	3.78 ± 0.93^ab^	91.963	< 0.001

Longitudinal changes in clinical scores were analyzed using LMMs, with time as a fixed effect and individual patient as a random effect to account for within-subject correlation and missing data. Variance components were estimated by REML. LSM with 95% CI at each visit are reported. Pairwise comparisons between time points were adjusted using the Bonferroni correction (adjusted α = 0.05/5 = 0.01)

^a^ Compared with baseline

^b^ Compared with the previous time point. All comparisons were adjusted using the Bonferroni method. *P* < 0.01 was considered statistically significant

^c^ Compared with the previous time point, *P* < 0.05

**Fig. 3:**
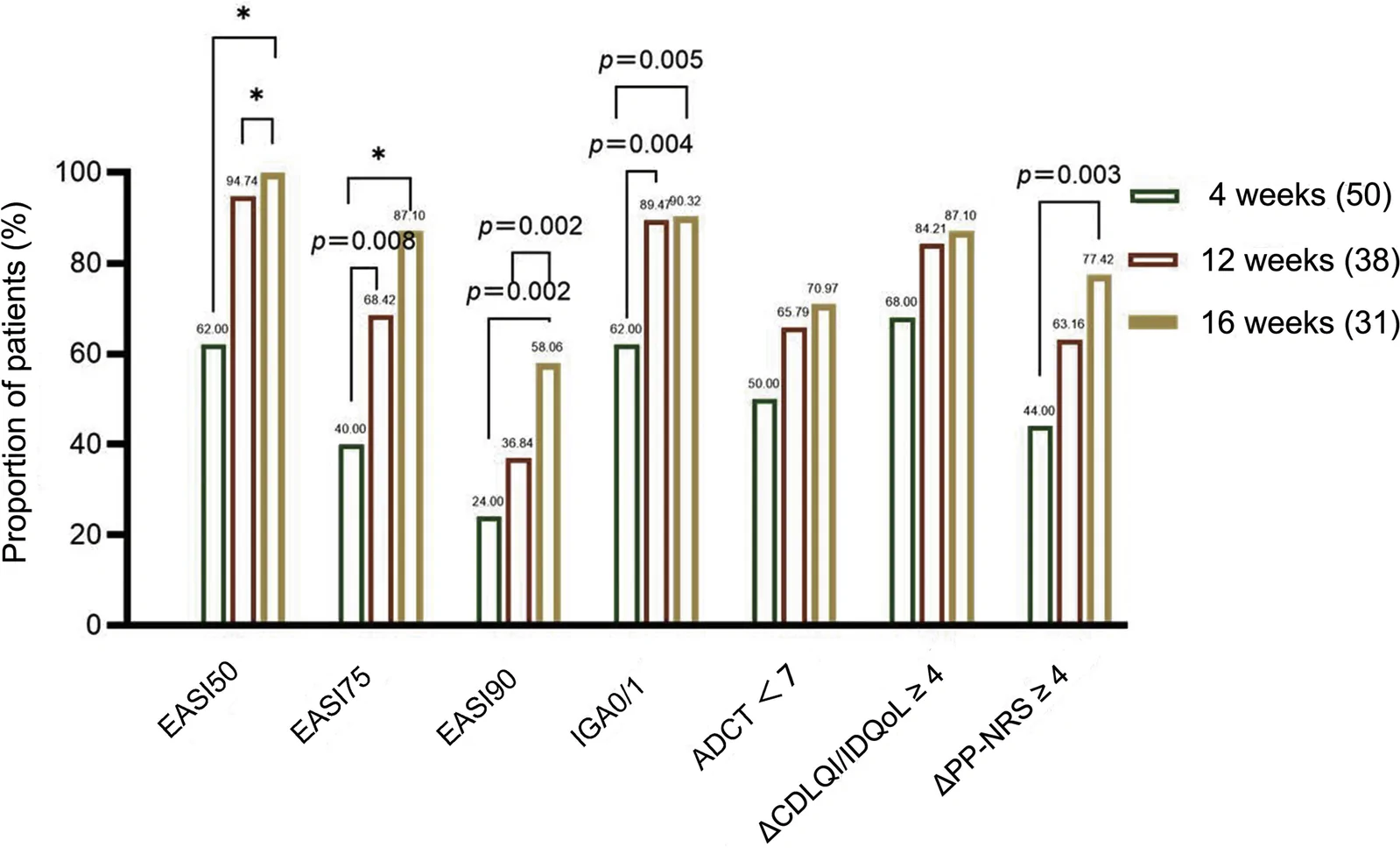
Proportions of children with AD achieving primary/secondary efficacy endpoints at different time points post-dupilimab treatment. Green: Week 4 (*n* = 50), Red: Week 12 (*n* = 38), Orange: Week 16 (*n* = 31). Proportions of patients achieving categorical efficacy endpoints at Weeks 4, 12, and 16 were compared using the χ² test with Bonferroni correction for pairwise comparisons (adjusted α = 0.05/3 ≈ 0.017). *: *P* < 0.001

**Fig. 4:**
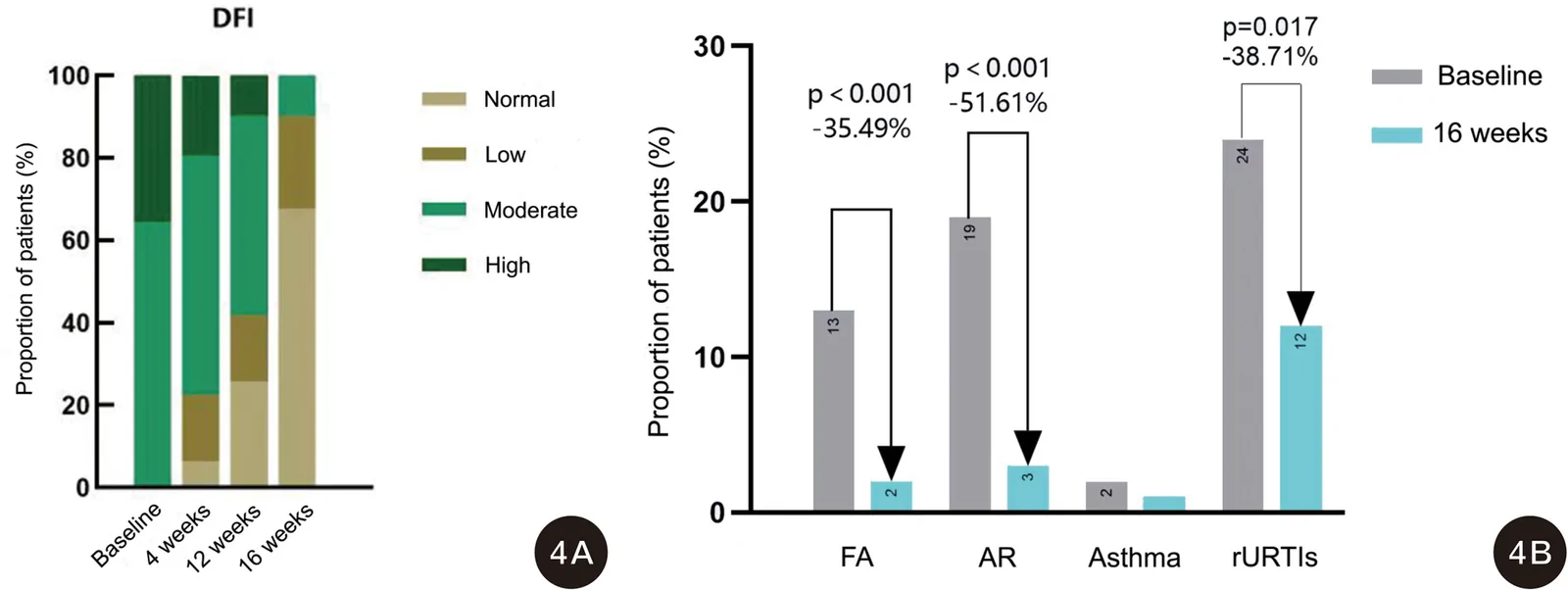
Changes in DFI severity and proportions of improved comorbidities/rURTIs in 31 children with moderate-to-severe AD after 16 weeks of dupilumab treatment (n/%). 4**A**: The DFI revealed that the proportion of caregivers with severe DFI impairment​ dropped from 35.48% (baseline) to 0 at Week 16, while those with moderate impairment​ decreased from 64.52% (baseline) to 9.68%. 4**B**: Significantly reduced proportions from baseline were observed: comorbid AR (-51.61%, *P*< 0.001); comorbid FA​ (-35.49%, *P*< 0.001); and rURTIs (-38.71%, *P*= 0.017). n=31. Categorical binary variables were compared using χ² test

Compared with those at week 4, the proportions of patients who achieved EASI50, EASI75, EASI90, IGA 0/1, or PP-NRS improvement ≥ 4 points were greater at week 16 (*P* < 0.05) (Fig. 3). At week 16 of treatment, the proportion of caregivers with moderate-to-severe DFI impairment decreased from 100% at baseline to 9.68% (Fig. 4A); the proportion of children with rURTIs decreased significantly (from 77.42% to 38.71%, *P* = 0.017); and improvements in allergic rhinitis and food allergy were observed in 84.21% and 84.61% of patients, respectively (both *P* < 0.001) (Fig. 4B).

### Baseline clinical characteristics associated with eSR

At week 4, 20 of 50 patients (40%) achieved eSR. To ensure model stability, multicollinearity diagnostics were performed for all variables. All variance inflation factors (VIFs) were below 5 (Table 4 for the exact values), indicating no evidence of significant multicollinearity. Univariate logistic regression revealed that onset before 6 months of age, allergic rhinitis, and rURTIs as well as higher absolute basophil counts, hemoglobin levels, mean corpuscular volume (MCV), and mean corpuscular hemoglobin (MCH) levels were potential predictors (*P* < 0.2). Variables found to be significant in univariate analysis were screened in accordance with the EPV criterion (≥ 10). As a result, two predictors were included in the final multivariable logistic regression model. a high absolute basophil count (OR = 0.104, 95% CI = 0.021–0.506, *P* = 0.005) and the presence of allergic rhinitis (OR = 0.223, 95% CI = 0.055–0.896, *P* = 0.035) were independent predictors of achieving eSR (*P* < 0.05).(Table 4). Bootstrap internal validation confirmed that the regression effects of both variables were robust and reliable (both *P* < 0.05).

**Table 4:** Binary logistic regression analysis of predictors of early super-response to dupilumab

Baseline characteristics	Multicollinearity	Univariate Logistic Regression			Multivariate Logistic Regression	
	VIF	M(Q1, Q3)/*n*%	OR(95%CI)	*P*	OR(95%CI)	*P*
Gender						
Female		29(58.00)	1			
Male	1.374	21(42.00)	0.815(0.259 ~ 2.559)	0.726		
Age	1.697	4.24 ± 1.81	0.839 (0.609 ~ 1.156)	0.282		
Onset ≤ 6 months of age	1.795	26(52.00)	0.444(0.140 ~ 1.411)	0.169		
Moderate AD	1.368	16(32.00)	1.167(0.344 ~ 3.956)	0.805		
Severe AD		34(68.00)	1			
Molecular endotype						
intrinsic AD	1.397	14(28.00)	1.286(0.358 ~ 4.617)	0.700		
extrinsic AD		36(72.00)	1			
rURTIs	1.241	30(60.00)	0.351(0.108 ~ 1.138)	0.081		
Allergic co-morbidities						
Allergic Rhinitis	2.109	23(46.00)	0.328(0.099 ~ 1.086)	0.068	0.223(0.055 ~ 0.896)	0.035
Food Allergy	1.654	16(32.00)	0.857(0.253 ~ 2.906)	0.805		
Family History of Atopy						
Allergic Rhinitis	2.052	24(48.00)	0.818(0.263 ~ 2.546)	0.729		
Eczema	1.403	10(20.00)	0.580(0.131 ~ 2.575)	0.474		
Breastfeeding	1.611	41(82.00)	0.800(0.186 ~ 3.434)	0.764		
Laboratory parameters						
Total serum IgE level	1.450	159.5(34.8, 611.5)	1.000(1.000 ~ 1.001)	0.600		
AEC	2.198	0.30(0.18, 0.66)	0.537(0.133 ~ 2.171)	0.383		
ABC	1.765	0.04(0.03, 0.06)	0.131(0.030 ~ 0.575)	0.007	0.104(0.021 ~ 0.506)	0.005
Neutrophil percentage		39.77 ± 15.24	0.994(0.957 ~ 1.033)	0.762		
Hemoglobin	1.469	131.26 ± 9.40	0.947(0.888 ~ 1.011)	0.102		
MCV	3.207	81.91 ± 3.48	0.867(0.723 ~ 1.041)	0.125		
MCH	3.684	27.41 ± 1.32	0.582(0.320 ~ 1.057)	0.076		

Univariate logistic regression was used for initial variable screening, and all variables with *P* < 0.2 were considered as candidates. Multicollinearity among these candidates was assessed using theVIF, with VIF > 10 indicating high collinearity; variables exceeding this threshold were excluded. The remaining predictors were then entered into a multivariable logistic regression model. To reduce overfitting, the final predictors were selected based on the EPV (EPV ≥ 10) principle, and bootstrap internal validation was performed to assess the robustness of the regression coefficients. Adjusted odds ratios are reported; natural log (Ln) transformation was applied for robust estimation where appropriate

### Predictive value of absolute basophil count for eSR

**Fig. 5:**
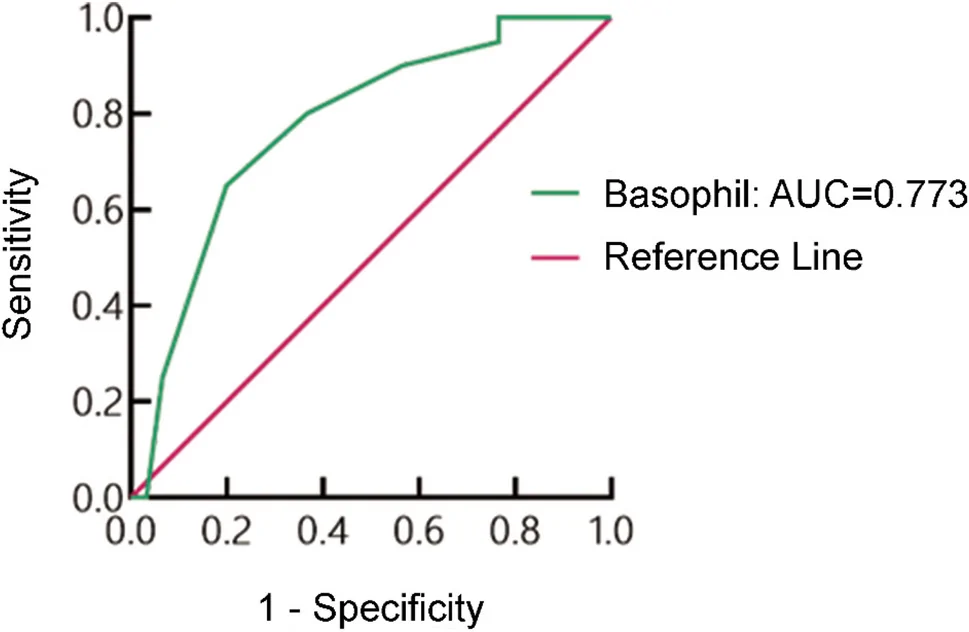
Predictive value of Absolute Basophil Count (ABC) levels in children with moderate-to-severe aD for eSR to dupilumab treatment

ROC curve analysis revealed an optimal cutoff value of 0.035 × 10⁹/L for the ABC to effectively predict eSR to dupilumab in children aged 6 months to 6 years with moderate-to-severe AD, yielding a sensitivity of 80.0% and a specificity of 65.0%. The area under the ROC curve (AUC) was 0.773 (95% CI: 0.641 ~ 0.906, *P* = 0.001), indicating moderate predictive accuracy. Notably, a significantly greater percentage of children with a baseline ABC below this cutoff had an eSR to dupilumab (Fig. 5).

Baseline ABC was used to construct a ROC curve; the Youden index was employed to determine the optimal cut-off, and corresponding sensitivity, specificity, and area under the curve (AUC) were reported. The ROC curve demonstrated that the AUC for predicting eSR using absolute basophil count was 0.773(*95% CI*: 0.641 ~ 0.906, *P* = 0.001).

### Incidence of adverse reactions

The most common treatment-emergent ADRs included one case of constipation and two cases of conjunctivitis, which typically resolved spontaneously within 1–3 days. One patient reported injection-site pain and erythema after each dupilumab administration, with symptoms resolving on the same day. No patients discontinued treatment because of injection-related adverse events, and no serious adverse events occurred throughout the study period.

## Discussion

Dupilumab, the first and only globally approved targeted biologic for moderate-to-severe AD across all age groups (from infancy to adulthood), exerts its therapeutic effect by specifically binding to IL-4Ra and inhibiting IL-4 and IL-13, thereby blocking critical inflammatory cascades. While its efficacy in adult populations has been well established, real-world data concerning the safety and efficacy of dupilumab in young children in China—particularly those aged less than 6 years—as well as the factors influencing early rapid/slow response remain scarce.

This study enrolled 50 pediatric patients at baseline, and 31 completed the 16-week follow-up, giving a dropout rate of 38%, indicating the presence of selection bias during follow-up. Comparison of baseline characteristics between the two groups showed no statistically significant differences in core indicators such as age, skin lesion severity (EASI, SCORAD, IGA), pruritus, and quality of life; differences were observed only in recurrent upper respiratory tract infections, allergic rhinitis, and baseline POEM scores. This may be because patients with these comorbidities or higher POEM scores were more willing to adhere to follow-up, whereas those with milder symptoms voluntarily discontinued clinic visits after early improvement. Although this study used a LMMs to handle missing data, the baseline imbalance caused by dropout remains a notable limitation, and further validation is needed through prospective cohort studies with larger sample sizes and improved follow-up management.

This study systematically evaluated the clinical efficacy and safety of dupilumab in children with moderate-to-severe AD aged 6 months to 6 years who were treated at the General Hospital of Ningxia Medical University. At week 16, after treatment with standard-dose dupilumab, the EASI75 response rate reached 87.10%, and the IGA 0/1 response rate reached 90.32%. These results are consistent with domestic and international real-world data. A retrospective real-world study from Spain involving 19 children under 6 years old with moderate-to-severe AD reported EASI75 and IGA 0/1 response rates of 84.2% (16/19) and 73.7% (14/19), respectively, at week 16 [22]. A retrospective Canadian study across 6 research centers, including 29 children under 6 years old with moderate-to-severe AD, revealed an EASI75 response rate of 90% (17/19) at week 16 [23]. Currently, domestic reports on AD in children under 6 years old are limited. A case report from Beijing involving 7 children aged < 6 years with AD who were treated with dupilumab reported an EASI75 response rate of 71.43% (5/7) at the treatment endpoint [24].

In the clinical management of pediatric AD, the DFI is regarded as a core patient-reported outcome (PRO) for assessing disease burden because it comprehensively captures the indirect impact of the disease on overall family well-being—particularly crucial for pediatric patients aged < 12 years ([25, 26]). High DFI scores are also significantly positively correlated with the severity of the child’s disease ([27, 28]). Our study demonstrated that after 16 weeks of dupilumab treatment, the percentage of caregivers with moderate-to-severe DFI impairment decreased from 100.00% at baseline to 9.68%, and CDLQI/IDQoL scores improved from 13.52 ± 6.05 to 4.13 ± 3.57 (*P* < 0.001). These findings suggest that dupilumab treatment is associated with a reduction in the multidimensional impact of AD on families, an improvement in children’s quality of life, and a significant alleviation of caregiver burden.

A retrospective Danish cohort study demonstrated that children with AD have a 1.2–1.8 times greater risk of upper/lower respiratory tract infections (rURTIs/LRTIs) than non-AD children do [29]. Tsai et al. conducted a 6-year cohort study involving 7,150 AD patients (< 18 years old) and reported that compared with other systemic therapies, dupilumab significantly reduced the risk of respiratory infections by 44% (RR = 0.56, 95% CI = 0.51–0.61) [30], with a more pronounced reduction in children aged 0–5 years. In our study, after 16 weeks of dupilumab treatment, the incidence of rURTIs in 31 children with AD decreased significantly from 77.42% at baseline to 38.71% (*P* = 0.017). This effect may be attributed to improved airway epithelial barrier dysfunction and modulation of the Th1/Th2 immune balance following dupilumab treatment [31], and further validation is needed in prospective controlled studies.

AD typically begins early in life. A multinational cross-sectional study (*n* = 7,465; 6 months–18 years) reported that 92.5% of children with AD had at least one coexisting type 2 inflammatory disease [32]. In our cohort, among 50 children with AD, 23 (46.00%) had comorbid allergic rhinitis (AR), 3 (6.00%) had asthma, and 16 (32.00%) had FA; additionally, 8 (16.00%) had two or more comorbidities. Multiple studies have confirmed that AD shares pathophysiological mechanisms centered on type 2 inflammation with atopic comorbidities such as AR, asthma, and FA [33]. Thus, clarifying the intervention efficacy of dupilumab for AD and its comorbidities is critical for advancing treatment progress.

A 3-year cohort study involving 4,384 pediatric patients (≤ 18 years old) in Taiwan, China, demonstrated that dupilumab significantly reduced the risk of atopic march progression, with the greatest benefit observed in children under 6 years of age (HR = 0.537, 95% CI: 0.381–0.756). Additionally, a 31% reduction in AR risk was detected in pediatric AD patients treated with dupilumab [34]. In our study, after 16 weeks of treatment, the rates of improvement in AR and FA were 84.21% and 84.61%, respectively, in children with AD. Among 2 children with comorbid asthma, 1 showed improvement in asthma symptoms. These findings suggest that during the blockade of the IL-4Rα pathway by dupilumab, alleviation of AD symptoms and concurrent improvement in comorbidities are observed. However, given the lack of a control group and the limited follow-up duration, these improvements cannot be directly attributed to dupilumab treatment; changes in food allergy are particularly susceptible to confounding factors. The independent therapeutic effect of dupilumab on allergic comorbidities still requires validation in long-term controlled studies.

The safety profile revealed that the overall incidence of treatment-emergent adverse events in this study was 8.00% (4/50). The most frequently observed events included conjunctivitis (4.00%, 2/50), constipation (2.00%, 1/50), and local injection-site reactions (2.00%, 1/50). These findings are consistent with those of a prior Chinese study involving 136 children and adolescents that reported an overall adverse event rate of 3.3%, including one case of conjunctivitis [35]. In alignment with the literature, adverse ocular events—primarily conjunctivitis—were the most common treatment-related findings, with a reported incidence of 10.17% in previous reports [36]. Notably, constipation was observed as an infrequent adverse event in the present cohort, which has rarely been documented in pediatric dupilumab studies. A real-world study in China reported a 5.51% incidence of gastrointestinal adverse events (e.g., abdominal pain and bloating) [37]. In our study, a single case of mild constipation was reported, which resolved spontaneously without interruption of treatment. The variation in incidence may be influenced by regional dietary patterns and gut microbiota profiles; however, the low frequency suggests no substantial divergence from other studies. Injection-site reactions were also consistent with known patterns. All documented adverse events were mild and self-limiting, and none resulted in treatment discontinuation, supporting the favorable safety profile of dupilumab in young pediatric patients.

Although dupilumab demonstrates favorable clinical efficacy in the management of pediatric AD, variability in early response speed persists among some children. The concept of eSR was initially derived from biologic therapy for psoriasis, characterized by rapid and sustained complete skin clearance. This concept has been substantiated by multiple studies and carries profound prognostic significance [38]. With the application of targeted therapies in AD, Nettis et al. first defined eSR in adult patients with moderate-to-severe AD in 2021 as those who simultaneously met the criteria of EASI 75, PP-NRS ≥ 4, and the Dermatology Life Quality Index (DLQI) ≥ 4 at week 4 of dupilumab treatment, and they employed this composite endpoint as an indicator to explore predictors of treatment response [9]. This composite endpoint encompasses the three domains recommended by the Harmonising Outcome Measures for Eczema (HOME) core outcome set—clinical signs, pruritus, and quality of life—and, compared with EASI 75 alone, is better able to identify pediatric patients who achieve multidimensional improvement in the short term [39]. The present study adopted this definition.

In this study, 20 out of 50 patients (40.00%) achieved eSR at week 4 of treatment. Univariate logistic regression analysis revealed the following potential predictors of eSR: onset before 6 months of age, AR, comorbid rURTIs, and elevated absolute basophil counts, hemoglobin levels, MCV and MCH levels (*P* < 0.2).

Multivariate logistic regression analysis revealed that a high absolute basophil count (*P* = 0.005) was independent predictors of eSR. Notably, this key conclusion has not been reported in similar studies so far. In a cohort of 388 adults with moderate-to-severe AD, Nettis et al. reported that no prior history of allergic conjunctivitis and history of allergic asthma were independent predictors of eSR to dupilumab. Another retrospective study involving 117 adults with severe AD reported that early-onset AD (onset < 18 years) combined with the absence of eosinophilia might serve as a predictive biomarker for early treatment response to dupilumab [9, 40]. A real-world cohort study in China enrolling 233 patients with atopic dermatitis (AD) showed that, on univariate analysis, elevated baseline serum total IgE levels were associated with poor response to dupilumab at week 4 of treatment [41]. Variations in the predictors of early response across studies [35] may be attributed to differences in sample size, patient age, disease severity, or comorbidities, necessitating larger-scale studies for further validation.

Further ROC curve analysis revealed that an absolute basophil count less than 0.035 × 10⁹/L exhibited moderate discriminatory performance for predicting eSR to dupilumab (AUC = 0.773; 95% CI = 0.641–0.906), with a sensitivity of 80.0% and a specificity of 65.0%. In AD, the increased expression of the basophil activation marker CD203c indicates the involvement of basophils, as also demonstrated by Hu et al. ([42, 43]). Thymic stromal lymphopoietin (TSLP) and IL-33 released upon skin barrier disruption activate basophils, inducing them to secrete large amounts of IL-4 ([44, 45]). IL-4 drives B cells to produce IgE, which binds to the high-affinity IgE receptor (FcεRI) on basophils and becomes cross-linked, in turn enhancing basophil activation and IL-4 release. IL-4 also promotes ILC2s to secrete IL-5 and IL-13, which drive eosinophil differentiation and chemotaxis [46]. Infiltrating eosinophils release factors such as IL-4, which not only aggravate tissue damage but also re-activate basophils. It is therefore speculated that IgE and eosinophils are essentially downstream effectors and amplification products of basophil-mediated responses. Further studies suggest that basophils exhibit reduced expression of S1PR1 [47]. Sphingosine-1-phosphate (S1P) is a lipid mediator that binds to S1PR1 to participate in lipid signaling and keratinocyte regulation. In patients with atopic dermatitis, decreased S1PR1 expression attenuates the effects of S1P, thereby aggravating the disease. Basophils can also amplify itch signals by releasing lipid mediators such as leukotriene C4 or by directly interacting with sensory nerve endings, thereby establishing an “itch–scratch” cycle [48].

Moreover, AR is an independen predictor of eSR in pediatric AD. This study enrolled children aged 6 months to 6 years, previous studies have shown that AR in preschool children warrants clinical attention: cross-sectional studies in China and Thailand reported mean ages of onset of 4.7 ± 1.2 years and 4.37 ± 0.76 years, respectively, with prevalence ranging from 2.8% to 42.6% ([49, 50]), suggesting that AR is relatively uncommon in younger children and more prevalent in older children. To control for confounding, we performed collinearity diagnostics for all variables; after excluding variables with VIF > 10, all remaining VIFs were < 5, indicating no significant collinearity between age and AR. It should be noted that this conclusion may be limited by the relatively small sample size, and the multivariable logistic regression model may be subject to potential instability, future studies with larger samples are warranted to validate. Based on the above analyses, multivariable logistic regression showed that AR is an independent predictor of eSR in children with AD.

AD, AR, and asthma are collectively termed the “atopic triad,” sharing a systemic type 2 inflammatory burden. Epidemiological studies show that approximately two-thirds of AD patients progress to AR, with earlier AD onset correlating with a higher subsequent risk of AR. Patients comorbid with AR and/or asthma have a 5.88-fold higher risk of progressing to moderate-to-severe AD compared with those without atopic diseases ([51, 52]). This comorbid state may impair treatment response through the cumulative effect of multi-organ inflammatory networks: disruption of the nasal mucosal barrier in AR patients facilitates easier allergen penetration, amplifying type 2 inflammatory cascades centered on IL-4/IL-13. A meta-analysis involving 5,575 AD patients (including children and adults) revealed that no prior history of AR was significantly associated with good treatment response to dupilumab (at 12–16 weeks) [53].

A retrospective RCT in China involving 74 children under 6 years of age who received a high-dose loading regimen (initial dose doubling) revealed that at week 2 of treatment, the EASI75 response rate with the loading dose was 17.4%, and the IGA 0/1 response rate was 30.40%. In contrast, the corresponding rates with the standard dose were 0% and 2.9%, respectively. The percentage of patients who achieved a ≥ 4-point improvement in the pruritus NRS score was significantly greater in the high-loading dose group (69.6%, 16/23) than in the standard-loading dose group (23.5%, 8/34) (*P* < 0.001). Moreover, compared with the standard regimen, the high-loading dose regimen did not significantly increase the incidence of adverse events [54].

These findings may suggest that for pediatric patients under 6 years of age, an initial high-dose loading regimen could more effectively inhibit the development of Th2-type inflammation and achieve rapid symptom control. On the basis of the abovementioned mechanisms and clinical evidence, future studies may explore the effects of increasing the initial treatment dose or shortening the dosing interval for AD children with elevated blood basophil counts and/or comorbid AR. This approach aims to block the synergistic amplification of type 2 inflammation and improve the early rapid response rate.

Several limitations of this study should be considered. First, the single-center, noncontrolled design and relatively limited sample size of this study, which was conducted at a tertiary hospital in Northwest China, may introduce selection bias and restrict the generalizability of the findings. Therefore, further validation through multicenter studies with larger cohorts is warranted. Second, the relatively brief follow-up period of 16 weeks, which may have introduced some degree of follow-up bias, may be insufficient to fully evaluate the long-term efficacy and safety profile of dupilumab in pediatric patients with atopic dermatitis; extended observational studies are needed to provide more comprehensive evidence. Third, partial loss to follow-up occurred because of suboptimal compliance in some participants, which might have affected the completeness and accuracy of the results.

## Conclusion

In conclusion, this study revealed that dupilumab significantly improved symptoms in children aged 6 months to 6 years with moderate-to-severe AD. After 16 weeks of treatment, favorable efficacy and safety were observed, with the treatment effectively increasing children’s quality of life. During the follow-up period, the burden associated with allergic comorbidities and respiratory infections was also alleviated. Future studies may optimize dosing regimens on the basis of basophil levels and allergic rhinitis comorbidity to develop personalized treatment strategies. Nevertheless, large-scale cohort studies are still needed to further validate the application of dupilumab in young children. 

## Supplementary Information

Supplementary Material 1.

Supplementary Material 2.

## Data Availability

The datasets generated and/or analyzed during the current study are not publicly available due to patient privacy and institutional data protection regulations, but are available from the corresponding author on reasonable request with ethical approval.

## Abbreviations

AD: Atopic Dermatitis
AR: Allergic Rhinitis
EASI: Eczema Area and Severity Index
SCORAD: SCORing Atopic Dermatitis
IGA: Investigator’s Global Assessment
PP-NRS: Peak Pruritus Numeric Rating Scale
CDLQI: Children’s Dermatology Life Quality Index
IDQoL: Infant’s Dermatology Life Quality Index
ADCT: Atopic Dermatitis Control Tool
DFI: Dermatitis Family Impact
RCAT: Rhinoconjunctivitis Control Assessment Test
C-ACT: Child Asthma Control Test
OFC: Oral Food Challenge
POEM: Patient-Oriented Eczema Measure
rURTIs: Recurrent upper respiratory tract infections
eSR: Early Super-Responder
