# Supplementary material for: Dupilumab efficacy in children aged 6 months to younger than 6 years with moderate-to-severe atopic dermatitis and clinical characteristics of early super-responders
Source: BMC Pediatr. 2026 Jul 22;26:1028. doi: 10.1186/s12887-026-07381-6 (PMC13685078; doi:10.1186/s12887-026-07381-6)
Supplement: Supplementary file 1 [file 12887_2026_7381_MOESM1_ESM.pdf]

宁夏医科大学总医院医学科研伦理审查委员会批准书

项目名称	度普利尤单抗治疗儿童中重度特应性皮炎疗效、安全性及影响疗效因素预测：一项真实世界研究				伦理编号	KYLL-2024-1447	
项目负责人	尚元元	职称	主任医师	联系电话	13709589968	科室	皮肤科
负责人研究单位：		宁夏医科大学总医院					
合作研究单位：							
研究时间：		2023-09-01 至 2025-10-01					
是否使用人体组织标本和样本		是					
试验数量：		20					
是否涉及干细胞与再生医学		否					
研究项目来源：		其他					
其他伦理相关文件							
是否附有知情同意书：		1					
获取知情同意书方法：		适当					
附件上传：							
知情同意书		知情同意书1.pdf					
试验方案		研究方案.pdf					
技术路线图		技术路线图.pdf					
幻灯PPT		PPT.pdf					
研究材料诚信承诺书		科研诚信承诺书.pdf					
生物样本信息数据的来源证明							
利益冲突申明							
招募广告及其发布形式							
审查形式		01					
年度/定期跟踪审查频率		半年					
评审意见：		符合伦理学要求，可以按照此方案进行试验。					

医学科研伦理委员会审核通过

宁夏医科大学总医院医学科研伦理审查委员会<br>审批时间：2024年09月14日	
委员会审批意见	王幽深 【通过】<br>朱禧 【通过】<br>杨笑 【通过】<br>刘丹 【通过】<br>郭若冰 【通过】<br>丁晓云 【通过】<br>赵巍 【通过】<br>朱力 【通过】<br>陈娟 【通过】

医学科研伦理委员会审核通过
