# Supplementary material for: Dupilumab efficacy in children aged 6 months to younger than 6 years with moderate-to-severe atopic dermatitis and clinical characteristics of early super-responders
Source: BMC Pediatr. 2026 Jul 22;26:1028. doi: 10.1186/s12887-026-07381-6 (PMC13685078; doi:10.1186/s12887-026-07381-6)
Supplement: Supplementary file 2 [file 12887_2026_7381_MOESM2_ESM.pdf]

Editing Certificate

This document certifies that the manuscript

Dupilumab efficacy in children aged 6 months to younger than 6 years with moderate-to-severe atopic dermatitis and clinical characteristics of early super-responders

prepared by the authors

Li Ma, Wenjia Mou, Li Xia, Nan Yu, Miaomiao Zhao, Peiyao Yu, Xiaoyu Jia, Yuanyuan Shang

was edited for proper English language, grammar, punctuation, spelling, and overall style by one or more of the highly qualified English speaking editors at AJE.

This certificate was issued on **November 21, 2025** and may be verified on the AJE website using the verification code **3607-5DC6-7EC5-BFB1-8AB8**.

Neither the research content nor the authors' intentions were altered in any way during the editing process. Documents receiving this certification should be English-ready for publication; however, the author has the ability to accept or reject our suggestions and changes. To verify the final AJE edited version, please visit our verification page at aje.com/certificate. If you have any questions or concerns about this edited document, please contact AJE at support@aje.com.

AJE provides a range of editing, translation, and manuscript services for researchers and publishers around the world. For more information about our company, services, and partner discounts, please visit aje.com.
